# Facing the crisis in human resources for eye health in sub-Saharan Africa

**Published:** 2018-02-08

**Authors:** Ronnie Graham

**Affiliations:** 1 Independent Consultant, Lower Largo, Scotland.

**Sub-Saharan Africa does not yet have enough eye health workers to help the millions of people suffering from eye disease in this region. This article explains the challenges in sub-Saharan Africa and the efforts underway to train and empower more eye health workers.**

**Figure:**
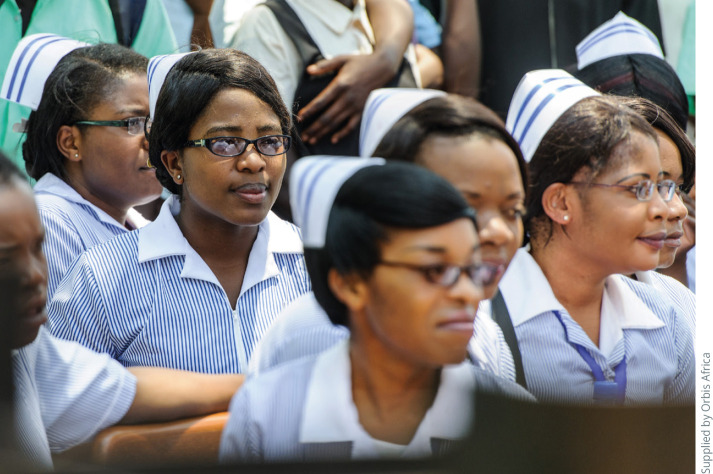
Ophthalmic nurses attend World Sight Day celebrations. SOUTH AFRICA

Eye disease in sub-Saharan Africa is now estimated to affect 18–25% of the population. This includes blindness (0.6%–1% of the population), moderate to severe visual impairment (3.6%–4%), presbyopia (7%–8%) and ‘all other ocular morbidities’, an overarching term that describes any significant eye condition which may or may not cause sight loss (8%–10%).

To deal with this total burden of eye disease, sub-Saharan Africa must establish comprehensive eye health services, available to all. This requires an eye health workforce capable of working as a team at community, primary, secondary and tertiary levels. Without such a workforce, it will not be possible to meet the need for eye care in sub-Saharan Africa.

Whereas some countries (e.g. Ghana and Kenya) are approaching the World Health Organization's (WHO'S) minimum requirements for eye health workers (Table 1), the majority of countries in sub-Saharan Africa remain under-resourced, particularly French and Portuguese speaking and conflict states (Table 2). The fact is that an insufficient number of new eye health workers are being trained for the needs of a growing and ageing population. Without government commitment to establish and fund professional pathways for eye care workers, the crisis is set to worsen.

**Table 1:** Minimum eye care team for 1 million population

Cadre	Key activities	Minimum per million population
Ophthalmologist	Diagnosis/treatment Eye surgery Training	4
Eye nurse/allied health personnel	Varies depending on training May work alone or with an ophthalmologist	10
Optometrist	Refraction, spectacles Screening for eye disease Low vision	10
Primary health care worker	Screens visual acuity Treats conjunctivitis Dispenses reading spectacles for people aged 50 and over	200

## International involvement

The importance of human resources for eye health (HReH) was first highlighted in 1999 with the launch of VISION 2020: The Right to Sight.^1^ The 2006 World Health Report, ‘Working Together for Health,’ renewed the emphasis on human resources.^2^

In 2016, the World Health Organization (WHO) launched Workforce 2030^3^, a new global strategy for human resources for health. It noted the need to increase the eye health workforce and urged member states to plan for the longer term. Workforce 2030 is a valuable resource for everyone involved in the health workforce arena.

There is a growing recognition that delivering comprehensive eye health, in a way that strengthens the health system^4^, will require sufficient people to fill each role within the eye health sector, and that they must have the training they need to do their job effectively. The WHO Global Action Plan for Universal Eye Health 2014–2019^5^ echoed this prioritisation with 3 out of 5 global indicators relating to the eye health workforce.

However, population growth, combined with an ageing population (who have a greater need for eye care) and the emerging problems of diabetes and myopia, means that the overall need for eye care is increasing.

## Achievements

Much has been achieved over the last two decades. The eye health sector has developed large HR projects:

- The Health for Peace initiative in West Africa (2006), a programme of training eye health workers for six small-population countries in West Africa
- DESSO, a francophone Diploma in Ophthalmology training programme set up in Guinea in 2006
- The East African College of Ophthalmology (EACO) was formed in 2010 and became the College of Ophthalmology of Eastern, Central and Southern Africa (COECSA)
- Ten new schools of optometry opened in Eritrea, Mali, Cameroon, Malawi, Zambia, Mozambique, Kenya, Uganda, and Zimbabwe (2008 onwards).
- Training for AOPs in Mozambique, Malawi and Zimbabwe was launched in 2009.

Additional activity by a number of international organisations, including members of the International Association for the Prevention of Blindness (IAPB), the Queen Elizabeth Diamond Jubilee Trust and the UK VISION 2020 LINKS Health Partnerships, have been of considerable benefit to individual training institutions and individual eye care workers.

## Advocacy and integrated planning

International non-governmental organisations (INGOs) have and can play a significant role in supporting the development of HReH, in particular by influencing governments to take more responsibility in line with the recommendations of Workforce 2030 and the African Platform for Human Resources for Health.

Integrated health workforce planning using the Workload Indicators of Staffing Needs (WISN) planning tool^6^ has now been implemented in Kenya, Malawi, Mozambique, Zambia, Cameroon and Ethiopia. WISN, which comes with its own computer software, is the workforce planning tool of choice. In Africa, the WHO Human Resources for Health Unit has introduced WISN to over 25 countries.

**Figure 1:**
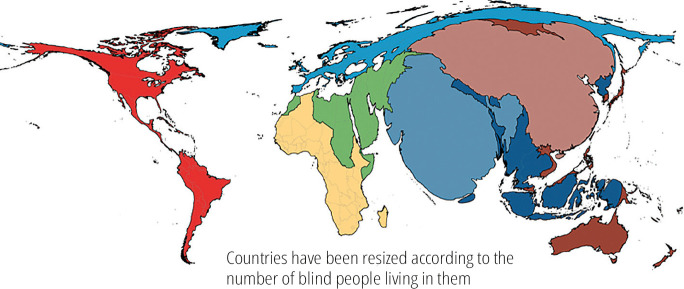
Where blind people live*

**Figure 2:**
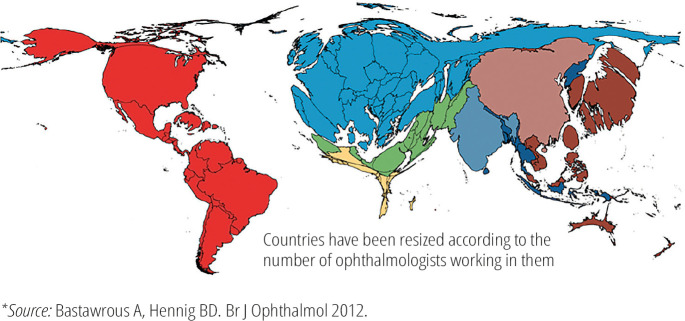
Where ophthalmologists work*

*Source: Bastawrous A, Hennig BD. Br J Ophthalmol 2012.

Governments have not historically prioritised eye health. However, they invest heavily in the health workforce (40% of health budgets), thereby providing an opportunity to integrate eye health into workforce planning.

## Ophthalmology

In 2016 and 2017, two expert meetings of the six colleges of ophthalmology in Africa, plus the Francophone Society, were convened with the objective of developing a harmonised, competency-based curriculum by 2020 and strengthening sub-specialty training in Africa.

## Optometry

Since 2006 ten new schools of optometry have been opened in Africa, but more remains to be done, particularly in Francophone Africa. Optometrists have a critical role to play given the magnitude of presbyopia and myopia.^7,8^

## Ophthalmic nurses and allied ophthalmic personnel

Ophthalmic nurses and allied ophthalmic personnel (ophthalmic clinical officers, ophthalmic assistants, etc.) are critical for eye care as the mid-level workforce. Their job titles, roles and responsibilities vary between countries, and issues of accreditation can limit personal and professional development. However, given the low number of eye specialists available, the mid-level workforce have an essential role in the eye care team. This must be recognised so that the best use can be made of their skills and availability.

## The eye health component of primary health care

The new WHO-AFRO Primary Eye Care training package (with algorithms) is in the process of being finalised and published. Its development was informed by various workshops, expert meetings and rigorous field testing in Rwanda and Kenya. Arguably, in terms of patients treated, this will do more to strengthen eye health systems and enhance coverage than any other development.

Strengthening community health workers was the key recommendation of WHO'S third global forum in 2013. This was echoed by the Policy Brief from WHO-AFRO (2017) and the survey of eye health and community health workers published by IAPB in 2015. The key challenge is to ensure that a comprehensive module for eye health is available at the country level so that the ‘bottom of the eye health pyramid of care’ is fully developed and available everywhere.

## Core competencies

Despite an increase in the different types of allied health personnel, only ophthalmologists, ophthalmic nurses, optometrists, opticians and orthoptists are specifically recognised in the current International Standard Classification of Occupations (ISCO-08). The core competencies of three key professionals in the eye health team (ophthalmologists, optometrists and allied ophthalmic personnel) are currently being validated by WHO-AFRO to provide technical guidance to training institutions and ministries of health and as the basis for curriculum review and the expansion of competency-based education. Recognition of new kinds of eye care personnel is necessary to ensure that the eye health workforce is recognised, rewarded and supported to address the crisis in service delivery.

**Table 2:** Distribution of eye health workers in by country language in sub-Saharan Africa

	Minimum required per million population (see Table 1)	Actual number per million population			
		Anglophone (English speaking)	Francophone	Lusophone	Total for sub-Saharan Africa
Population		574 million	281 million	53 million	908 million
Ophthalmologists (per million)	**4**	2.4	2.1	0.9	2,038
Optometrists (per million)	**10**	12.7	0.5	1.1	7,529
Ophthalmic nurses and Allied ophthalmic personnel (minimum 10 per million)	**10**	7.1	4.6	3.8	5,561
*Derived from Resnikoff, BJO, 2012, Palmer J., ICEH, 2014 and the IAPB Africa data base, January 2016.*					

## Priorities

### Strategic advocacy

In order to address the current crisis in service delivery, and ensure sustainable eye health provision, governments must be willing to recognise the different types of workers needed (e.g. ophthalmic nurses, allied health personnel or optometrists). Advocacy is needed to persuade governments to set up training programmes, professional standards, career paths and salary structures for these workers-all of which are needed to retain workers and ensure they are deployed where they are needed.

### Distribution

Alongside an overall shortage of eye health workers, there are large inequalities in their distribution which lead to inequalities in access to services within countries.^9^ Of particular concern is the distribution and retention of eye health workers in rural areas.^10^

In Africa, inequalities in the distribution of eye health workers between countries is most marked with respect to differences between Anglophone, Francophone and Lusophone Africa (Table 2). WHO'S recommended minimum ratio of ophthalmologist per population is 1 ophthalmologist per 250,000 population by 2020. In sub-Saharan Africa, there is now 1 ophthalmologist per 446,000 population on average; however, the ophthalmologists are unevenly distributed.

### Data

Better data on HReH are needed for advocacy, policy and planning at national level. Eye health workforce data must be integrated into existing health management information systems.

The Universal Eye Health Global Action Plan currently contains three HReH indicators. The WHO-AFRO catalogue has 27 indicators, of which five relate to the workforce. However, important information on retention, distribution by sector and location, continued professional development, competency, primary eye care and community health workers, sub-specialties, training capacity, task sharing and productivity is not collected systematically. This highlights the pressing need to move towards a standardised data collection tool. One attempt to achieve this important objective is the IAPB Africa database (IADb), which has now been introduced in a number of countries in the region.

## Future challenges

Achieving Universal Eye Health will require an efficient and well-run eye health system which ensures that people can obtain the eye health services they need without suffering financial hardship. It also requires access to essential eye medicines and technologies, and enough well-trained and motivated eye health workers.

To achieve these long-term objectives, the eye health sector in sub-Saharan Africa must change the way it works. Instead of focusing just on blindness and disease control, the emphasis should be on meeting national needs by establishing a comprehensive health service that offers universal access to eye health. We must also develop and support people who are willing to challenge barriers to improvement in a strategic manner.

HReH in sub-Saharan Africa is an ongoing crisis. Action is needed urgently to close the gap between the need for eye care and what is available. Where appropriate, organisations and individuals involved in addressing this crisis must:

- Work collaboratively in multi-agency consortia
- Develop teaching faculty to enhance the quality of training
- Provide essential equipment to ensure that graduates become productive and efficient as quickly as possible
- Offer regular continued professional development to enhance skills and improve job satisfaction
- Establish new partnerships with a range of stakeholders such as training institutions, regional health authorities, professional bodies and ministries of health and education
- Carry out strategic advocacy that encourages integrated planning of the health and eye health workforce.
- Collect, and provide access to, reliable and timely data that can be used for evidence-based planning.

## Summary

Resolving the human resources crisis in Africa requires a range of interventions. We must accelerate efforts to train eye health workers to a high standard. Addressing other challenges, such as distribution, remuneration, recognition and retention require the involvement of governments; they cannot be resolved by international non-governmental organisations working on their own.
